# Heart failure with preserved ejection fraction (HFpEF) in type 2 diabetes mellitus: from pathophysiology to therapeutics

**DOI:** 10.1093/jmcb/mjac028

**Published:** 2022-05-03

**Authors:** Miyesaier Abudureyimu, Xuanming Luo, Xiang Wang, James R Sowers, Wenshuo Wang, Junbo Ge, Jun Ren, Yingmei Zhang

**Affiliations:** Cardiovascular Department, Shanghai Xuhui Central Hospital, Fudan University, Shanghai 200031, China; Department of General Surgery, Shanghai Xuhui Central Hospital, Fudan University, Shanghai 200031, China; Cardiovascular Department, Shanghai Xuhui Central Hospital, Fudan University, Shanghai 200031, China; Diabetes and Cardiovascular Research Center, University of Missouri Columbia, Columbia, MO 65212, USA; Department of Cardiology, Shanghai Institute of Cardiovascular Diseases, Zhongshan Hospital, Fudan University, Shanghai 200032, China; Department of Cardiology, Shanghai Institute of Cardiovascular Diseases, Zhongshan Hospital, Fudan University, Shanghai 200032, China; Department of Cardiology, Shanghai Institute of Cardiovascular Diseases, Zhongshan Hospital, Fudan University, Shanghai 200032, China; Department of Laboratory Medicine and Pathology, University of Washington, Seattle, WA 98195, USA; Department of Cardiology, Shanghai Institute of Cardiovascular Diseases, Zhongshan Hospital, Fudan University, Shanghai 200032, China

**Keywords:** type 2 diabetes mellitus, heart failure with preserved ejection fraction, pathophysiology, therapies

## Abstract

Type 2 diabetes mellitus (T2DM or T2D) is a devastating metabolic abnormality featured by insulin resistance, hyperglycemia, and hyperlipidemia. T2D provokes unique metabolic changes and compromises cardiovascular geometry and function. Meanwhile, T2D increases the overall risk for heart failure (HF) and acts independent of classical risk factors including coronary artery disease, hypertension, and valvular heart diseases. The incidence of HF is extremely high in patients with T2D and is manifested as HF with preserved, reduced, and midrange ejection fraction (HFpEF, HFrEF, and HFmrEF, respectively), all of which significantly worsen the prognosis for T2D. HFpEF is seen in approximately half of the HF cases and is defined as a heterogenous syndrome with discrete phenotypes, particularly in close association with metabolic syndrome. Nonetheless, management of HFpEF in T2D remains unclear, largely due to the poorly defined pathophysiology behind HFpEF. Here, in this review, we will summarize findings from multiple preclinical and clinical studies as well as recent clinical trials, mainly focusing on the pathophysiology, potential mechanisms, and therapies of HFpEF in T2D.

## Introduction

The prevalence of type 2 diabetes mellitus (T2DM or T2D) rose dramatically over the past decades, afflicting ∼8% adults globally (Ren et al., 2010; Lascar et al., 2018; Zhang et al., 2018). The ever-rising T2D prevalence imposes profound sequelae on the cardiovascular system (Ceylan-Isik et al., 2008; Yang et al., 2015; Tan et al., 2020; Ren et al., 2021). Cardiovascular disease (CVD) becomes the leading cause of death in T2D, with coronary artery disease (CAD) and ischemic cardiomyopathy being the main culprits (Jia et al., 2018; Tan et al., 2020; Jankauskas et al., 2021). In addition to CAD, small vessel disease and diminished cardiac capillary density also occur (Zhou et al., 2018).

T2D evokes unique changes in myocardium independent of classical risk factors, including CAD, hypertension, and valvular heart disease. This is a condition termed as diabetic cardiomyopathy (DCM) (Jia et al., 2018; Tan et al., 2020). Early-stage DCM is usually characterized by structural and functional abnormalities, including myocardial hypertrophy, stiffening, interstitial fibrosis, and diastolic dysfunction (Ren and Ceylan-Isik, 2004; Wold et al., 2005; Ren et al., 2010; Jia et al., 2018; Tan et al., 2020). Similar findings were observed in animal models of T2D, including compromised diastolic and systolic function accompanied by changes in cardiomyocyte function and intracellular Ca^2+^ (Wold et al., 2005; Li et al., 2006; Ghosh et al., 2007; Ren and Anversa, 2015; Wang et al., 2021).

Patients with T2D exhibit an increased risk of heart failure (HF). Of note, 25% of T2D patients exhibit various types of HF, including HF with preserved, reduced, and midrange ejection fraction (HFpEF, HFrEF, and HFmrEF, respectively), with mortality increased by 30%–50% and worsened prognosis (Cosentino et al., 2020). The two predominant types of HF in DCM are HFrEF and HFpEF, with concentric left ventricular (LV) remodeling and diastolic dysfunction. HFpEF accounts for approximately half of the HF incidence in T2D, characterized by a normal or near-normal LV ejection fraction (LVEF) ≥50% and exercise intolerance as the chief complaint (McDonagh et al., 2021). Other than T2D, various complications, including obesity, hypertension, dyslipidemia, renal disease, and atrial fibrillation, also closely correlate with HFpEF (Taqueti et al., 2018). Given the high prevalence and poor prognosis of HFpEF, intensive therapy is pertinent for the management of cardiovascular anomalies in patients with T2D (Lalic, 2021; Tadic et al., 2021). Nonetheless, current treatment modality remains dismal largely due to the poorly defined pathophysiology of HFpEF in T2D. In this review, we discuss potential pathophysiological mechanisms and treatment options for HFpEF in T2D patients. We will also summarize recent clinical trials.

## Epidemiology and prognosis of HFpEF in T2D

T2D plays an essential role in the onset of HFpEF (Lalic, 2021; Tadic et al., 2021). Compared with non-diabetics, the incidence and mortality of CVD and HF are much higher in T2D patients (Bullard et al., 2018; Xu et al., 2018). Patients with T2D initially present with normal systolic but progressively impaired diastolic function, indicative of HFpEF. Approximately 45% of T2D patients develop HFpEF, and the prevalence of co-morbid T2D is more abruptly increased in T2D patients with new-onset HFpEF (Patel et al., 2016).

T2D in combination with HFpEF greatly enhances CVD risk and mortality (Cavender et al., 2015). TOPCAT (LVEF > 40%) is a multicenter, randomized, double-blind, placebo-controlled study of microvascular complications in T2D patients, including neuropathy, nephropathy, and retinopathy (Sandesara et al., 2018; De Marco et al., 2021). According to the CHARM Trial, 3023 patients were defined as LVEF > 40%, with an average follow-up of 37.7 months. Patients with T2D display greater volume overload, more severe myocardial injury, higher body mass index, hypertension (especially systolic pressure), coronary microvascular dysfunction, and renal abnormality (McHugh et al., 2019). The I-PRESERVE Study with LVEF ≥ 50% revealed that HFpEF patients with T2D had higher rates of cardiovascular mortality, hospitalization, and all-cause mortality (MacDonald et al., 2008; Kristensen et al., 2017; Table 1). Overall, these clinical trials denote a close tie between HFpEF-associated mortality and T2D, favoring a positive correlation between T2D and HFpEF-associated morbidity and mortality. Possible contributions from T2D-induced cellular, molecular, and metabolic abnormalities are considered the major driving forces for the worsened HFpEF pathology.

**Table 1:** Earlier clinical trials of non-sodium–glucose co-transporter 2 (non-SGLT2) inhibitor medications in HFpEF patients.

Trial	Treatment	Participants	Primary outcomes/ endpoints	Follow-up (average)	Population	Reference
CHARM-P (NCT 00634712)	Candesartan vs. placebo	LVEF ≥ 50%	CVD; recurrent HFH	2.9 years	*n* = 1953	Lund et al. (2018)
PEP-CHF	Perindopril vs. placebo	Age ≥ 70 years; LVEF > 40%	All-cause mortality and HFH by quartile of NT-pro BNP	1.0 year	*n* = 375	Cleland et al. (2012)
I-PRESERVE (NCT 00095238)	Irbesartan vs. placebo	Age ≥ 60 years; LVEF > 45%	CVD, HFH, and all-cause mortality according to with or without T2D	4.1 years	*n* = 4128	Kristensen et al. (2017)
TOPCAT (NCT 00094302)	Spironolactone or placebo	Age ≥ 50 years; LVEF ≥ 45%	CVD death; cardiac arrest; HFH	3.3 years	*n* = 3445	Pitt et al. (2014)
PARAGON-HF (NCT 01920711)	Sacubitril–valsartan or valsartan	Age ≥ 50 years; LVEF ≥ 45%	CVD; HFH	26 months	*n* = 4800	Solomon et al. (2019)

CHARM-P, Candesartan in Heart Failure: Assessment of Reduction in Mortality and Morbidity; HFH, heart failure hospitalization; I-PRESERVE, Irbesartan in Heart Failure with Preserved Ejection Fraction; NT-pro BNP, N-terminal pro brain natriuretic peptide; PARAGON-HF, Prospective Comparison of ARNI with ARB Global Outcomes in Heart Failure with Preserved Ejection Fraction; PEP-CHF, The Perindopril in Elderly People with Chronic Heart Failure Trial; TOPCAT, Treatment of Preserved Cardiac Function Heart Failure with an Aldosterone Antagonist.

## Pathophysiology of HFpEF in T2D

**Figure 1:**
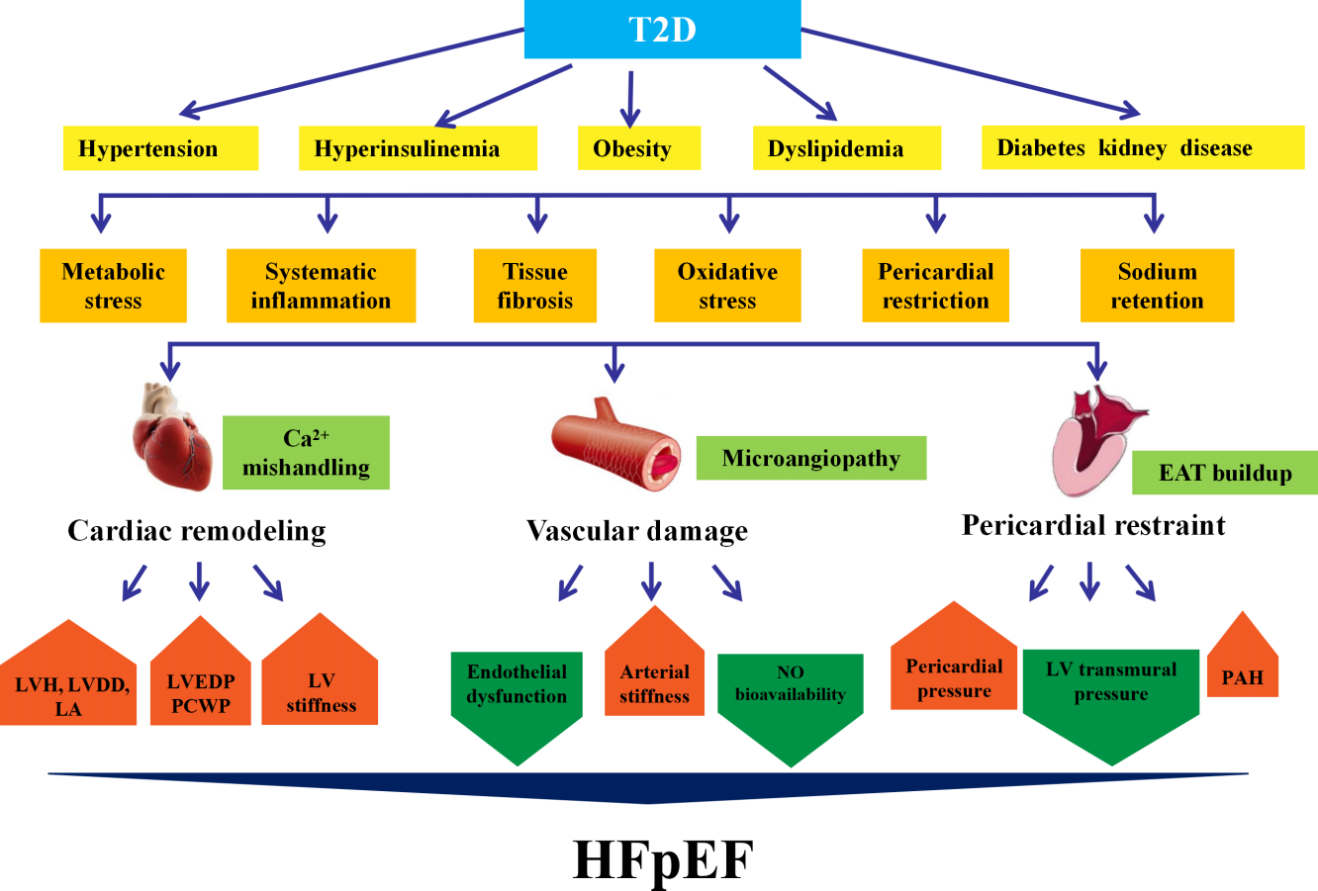
Pathology of HFpEF with T2D. Metabolic disturbances such as hyperglycemia, hypertension, hyperinsulinemia, obesity, and renal disease prompt the development of HFpEF in T2D. T2D patients with HFpEF often exhibit LVDD and small LV cavities with elevated LV filling pressures, as well as vascular damage, including endothelial and coronary microvascular dysfunction. T2D and obesity are intertwined and are accompanied by EAT buildup, deteriorating myocardial inflammation, and fibrosis. EAT leads to mechanical stress as a pericardial restraint for LV function. Abnormal hemodynamics between myocardium and pericardium increase pericardial pressure and LVEDP and reduce LV transmural pressure, resulting in higher pulmonary capillary pressure. LA, left atrium; PCWP, pulmonary capillary wedge pressure.

Clinical reports favor a DCM phenotype distinct from dilated cardiomyopathy. T2D patients display myocardial dysfunction in the absence of coronary heart diseases (e.g. CAD) (Dei Cas et al., 2015), possibly due to metabolic abnormalities, e.g. advanced glycation end product (AGE) deposition, lipid toxicity, and microvascular rarefication (Low Wang et al., 2016). These adverse effects of T2D were more pronounced in HFpEF (Lalic, 2021; Tadic et al., 2021). T2D with HFpEF is featured by LV diastolic dysfunction (LVDD) and decreased LV cavity with elevated LV filling pressure, as well as endothelial and coronary microvascular dysfunction (Paulus and Dal Canto, 2018). In T2D, hearts are exposed to hyperglycemic environments with abundant fatty acids and cytokines (Seferovic and Paulus, 2015; McHugh et al., 2019). Hyperglycemia provokes adverse clinical outcomes in T2D through AGE production (Ren and Ceylan-Isik, 2004), leading to accumulation of reactive oxygen species (ROS), inflammation, mitochondrial damage, and apoptosis (Ren and Ceylan-Isik, 2004; Wold et al., 2005). Hyperglycemia prompts endothelial dysfunction and elevated serum cholesterol, resulting myocardial dysfunction and ventricular–vascular uncoupling (Meza et al., 2019). In this context, subclinical variations develop, prompting cardiovascular events, including HFpEF (Figure 1; Kosmala et al., 2017). Rigorous glucose control is of enormous importance for the clinical outcome of HFpEF.

### LVDD

HFpEF in T2D is difficult to diagnose due to relatively mild and atypical symptoms, including poor exercise capacity (Stahrenberg et al., 2010). T2D can directly affect myocardial structure and function to prompt HFpEF (Mihara et al., 1989). T2D-induced specific cardiomyocyte anomalies include compromised Ca^2+^ handling, encompassing cytosolic and mitochondrial Ca^2+^ handling (Ren and Anversa, 2015). HFpEF is often manifested as diastolic dysfunction, with elevated LV filling pressure and plasma levels of BNP and NT-pro BNP (McMurray et al., 2012; Ejiri et al., 2020). The precise pathogenesis behind HFpEF is complex and multifactorial, with altered cardiac metabolism such as those seen in T2D being a critical contributing factor (Seferovic et al., 2018).

It is noteworthy that diastolic dysfunction occurs in T2D patients without obvious CAD symptoms. Three quarters of T2D patients may present diastolic dysfunction (Boyer et al., 2004), usually defined as defects in the integral of active diastolic relaxation dependent upon myofilament dissociation and cytosolic Ca^2+^ reuptake in early diastolic phase. Passive stiffness is related to viscoelasticity controlled by mechanical changes from sarcomere to extracellular matrix. T2D also induces ROS generation and deposition of AGEs in endothelial and smooth muscle cells, prompting concentric LV remodeling and wall stiffness (Lazo et al., 2015). Elevated serum AGEs were associated with prolongation of LV relaxation in early stage of DCM (van Heerebeek et al., 2008). In T2D, insulin resistant, and obese patients, circulating levels of glucose, free fatty acid (FFA), and proinflammatory cytokines were elevated, along with compromised angiogenesis, leading to pathological hypertrophy and diastolic dysfunction (Ren et al., 2021). The severity and duration of hyperglycemia function as an important determinant for the onset of LVDD. High FFA intake, for example, leads to lipid toxicity and accumulation of triglyceride (TG) in the myocardium. Cardiac steatosis, as visualized using proton magnetic resonance spectroscopy, denotes high TG content in the heart muscles to prompt diastolic defect (Ren et al., 2021). From these data, prominent changes in Ca^2+^ handling, deposition of AGEs, and insulin metabolic signaling may influence cardiac interstitial fibrosis/fiber stiffness and diastolic dysfunction in T2D patients with HFpEF.

### Endothelial dysfunction

Endothelial defect usually encompasses impaired vasodilation, enhanced vasoconstriction, arterial stiffness, and overt atherogenesis. In addition, signs of dyspnea and fatigue are also noted in association with submaximal exercise and altered bioavailability of nitric oxide (NO). Impaired insulin metabolic sensitivity is a vital pathophysiological abnormality tied to DCM, which inhibits endothelial NO synthase (eNOS) and NO production (Paulus and Tschope, 2013; Sickinghe et al., 2019; Nakamura and Sadoshima, 2020). The prevalence of endothelial dysfunction is high in HFpEF patients (Lam and Brutsaert, 2012). Endothelial dysfunction is linked to adverse outcomes in HFpEF patients, with a main role of poor NO availability (Paulus and Tschope, 2013). Compared with HFpEF patients without endothelial dysfunction in coronary arteries, patients with comprised coronary endothelial function possess more severe clinical outcomes associated with T2D. The main driving force for myocardial dysfunction in T2D includes insulin resistance and impaired glucose tolerance, long before the onset of full-blown T2D (Poornima et al., 2006). In addition, increased arterial stiffness and LV end-systolic stiffness or elasticity result in altered preload and afterload, ultimately leading to a major fluctuation of stroke volume (Seferovic et al., 2019). These changes help to explain the role of blood pressure instability in HFpEF patients. Exercise tolerance test suggests that patients with HFpEF may not get more benefits compared with those with HFrEF, courtesy of the loss of exercise benefit in the presence of endothelial dysfunction (Del Buono et al., 2019).

### Coronary microvascular dysfunction

The new focal point for HFpEF etiology has been shifted from LV volume overload to coronary microvascular inflammation. Coronary microvascular dysfunction is very difficult to define in HFpEF based on clinical indicators. In HFpEF, concentric LV remodeling is rooted from coronary microvascular endothelial inflammation in T2D (Paulus and Tschope, 2013). In contrast, eccentric LV remodeling in HFrEF is driven by cardiomyocyte death due to ischemia, viral infection, and lipotoxicity. HFpEF patients with endothelial-dependent microvascular dysfunction exhibit more severe diastolic defects and poor prognosis (Akiyama et al., 2012). In addition to compromised systolic reserve, insufficient vasodilation may be seen related to reduced LV end-systolic volume (LVESV) and elevated stroke volume in HFpEF. In HFpEF patients, the mean systemic vascular resistance and effective arterial elasticity usually drop, particularly during exercise (Borlaug et al., 2010). Elevated AGEs and hyperglycemia may prompt vascular damage in various vascular beds (Dhulekar and Simionescu, 2018). Microvascular remodeling and angiogenesis are important regulatory mechanisms in coronary vessels, affecting vascular function (Pries et al., 2015). It is perceived that decreased NO levels contribute to endothelial-dependent coronary micro-vasodilatation defects, causing vessel dilatory dysfunction in T2D.

### Ventricular–vascular uncoupling

With concurrent T2D and HFpEF, endothelial dysfunction indecently disrupts ventricular–vascular uncoupling and contributes to pathological changes in T2D with HFpEF (Lalic, 2021; Tadic et al., 2021). Systolic dysfunction often leads to subsequent impairment of LV diastolic function. Meanwhile, the interplay between vascular stiffness and diastolic reserve plays a major role in the etiology of HFpEF (Leite-Moreira et al., 2012). Ventricular load also seems to be important, with increased late systolic load exerting a greater adverse effect on LV diastolic function in comparison with increased early systolic pressure (Chirinos et al., 2013). The inability to dilate blood vessels with poor systolic reserve results in a dynamic limitation of ventricular the artery coupled with exercise intolerance in HFpEF patients (Phan et al., 2009).

### Diabetic kidney disease

Chronic end-stage renal disease, defined as diabetic kidney disease (DKD), is seen in ∼50% of T2D patients (Rangaswami et al., 2019). DKD contributes to secondary hypertension and anemia (Braunwald, 2019). In T2D, hyperglycemia leads to altered systemic metabolism. Cell stresses, including oxidative stress, endoplasmic reticulum (ER) stress, AGE buildup, inflammation, and histone and chromosomal abnormalities, all contribute to the etiology of DKD (Sakashita et al., 2021). Chronic renal dysfunction is related to increased hospitalization and death in HFpEF (Ananthram and Gottlieb, 2021). Consequently, T2D, kidney dysfunction, and HF are suggested to form a ‘vicious circle’ (Yamazaki et al., 2021). Decreased renal perfusion and elevated central venous pressure are the most important hemodynamic factors for the heart–kidney interplay. Moreover, the renin–angiotensin–aldosterone system and sympathetic nervous system are overactivated in T2D, leading to lipolysis and the onset of insulin resistance (Ren et al., 2021). Reduced sodium excretion promotes a proinflammatory state. This proinflammatory environment perpetuates a vicious cycle between the heart and kidney in HFpEF (Kashiwagi et al., 2021).

### Pericardial restriction

T2D is often concurrent with obesity to evoke metabolic changes, inflammation, fibrosis, and myocardial stiffness, all considered HFpEF phenotypes (van Woerden et al., 2018; Elsanhoury et al., 2021). One of the hallmarks of T2D and obesity is accumulation of epicardial adipose tissue (EAT), a form of endocrine tissues capable of deteriorating myocardial inflammation and fibrosis via the release of paracrine and autocrine factors (Mouton et al., 2020). Furthermore, enlargement of EAT leads to mechanical stress on pericardial restriction (Elsanhoury et al., 2021). In HFpEF, EAT thickness is closely associated with LV hypertrophy (LVH). Abnormal hemodynamics between myocardium and pericardium yields elevated pericardial pressure and LV end-diastolic pressure (LVEDP) and decreased LV transmural pressure that may elevate pulmonary capillary pressure. Finally, increased EAT volumes might also directly influence cardiac function and hemodynamics through mechanical interaction. Patients with HFpEF, particularly for obese HFpEF, exhibit an increased EAT thickness in collaboration with higher cardiac filling pressure and pericardial restraint, leading to pericardial constriction (Elsanhoury et al., 2021; Tschope et al., 2021; Ayton et al., 2022). With the escalating pericardial constraint, intracavitary diastolic filling pressure needs to be higher to achieve an adequate preload volume, which further increases pulmonary capillary hydrostatic pressures in patients with HFpEF. These structural changes may probably be resulted from inflammation evoked by adipokines. Moreover, excessive collagen deposition, abnormal protein glycosylation, and abnormal collagen cross-linking in the myocardium lead to increased LV filling pressure, decreased diastolic compliance, and an increased risk for development of HFpEF (Elsanhoury et al., 2021; Ayton et al., 2022).

## Molecular mechanisms behind HFpEF in T2D

**Figure 2:**
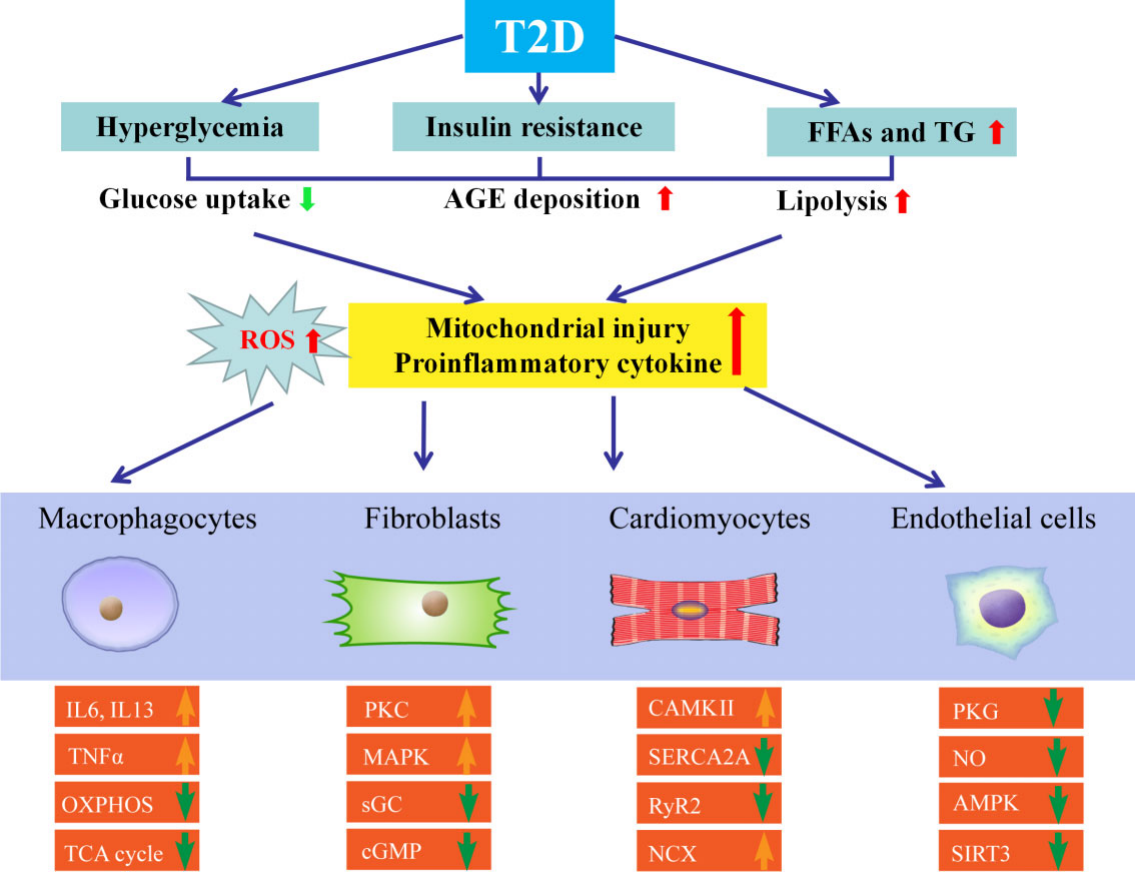
Molecular mechanisms behind HFpEF with T2D. Various metabolic abnormalities in T2D play an essential role in conjunction with the buildup of proinflammatory cytokines and ROS. Other confounding factors, including hyperglycemia, lipotoxicity, and increased levels of FFA, insulin, and AGEs, also contribute to HFpEF. Compromised NO bioavailability and sensitivity, oxidative stress and inflammation, and impaired angiogenesis are involved. Crucial molecular contributors to pathological hypertrophy include G protein-coupled receptors, stress hormone ligands, and signaling kinases in T2D with HFpEF. Relaxation period includes active and passive phases. Downregulated insulin signaling is a hallmark of T2D along with changes in other signaling cascades, including downregulated AMPK signaling and hyperactivated PKC and MAPK. IL6, interleukin 6; IL13, interleukin 13; OXPHOS, oxidative phosphorylation; TCA, tricarboxylic acid cycle; TNFα, tumor necrosis factor-α.

In T2D patients, decreased diastolic function accompanied by altered protein expression governing relaxation was revealed. Metabolic abnormalities in T2D seem to play a vital role in conjunction with hyperglycemia, proinflammatory responses, and lipotoxicity (Borlaug, 2014). Other confounding factors, including interstitial fibrosis, vascular damaging, dysregulated NO, and cyclic guanine monophosphate (cGMP), may also contribute to the etiology of HFpEF. For example, interruption in the cGMP–PKG signal transduction and the rise of protein kinase C alpha (PKCα) activity are deemed key regulatory factors of cardiomyocyte stiffness in DCM (Figure 2).

LVH is a cardinal feature of HFpEF and the major reason for elevated diastolic filling pressure and diastolic dysfunction. Myocardial fibrosis, impaired Ca^2+^ handling, oxidative stress, mitochondrial dysfunction, and metabolic reprogramming are all key components of pathological cardiac hypertrophy (Liang et al., 2021; Ren et al., 2021; Xu et al., 2021; Zhang et al., 2021). Crucial molecular contributors to this pathological hypertrophy in T2D with HFpEF include G protein-coupled receptors, stress hormone ligands, and signaling kinases (Nakamura and Sadoshima, 2018). The precise roles of these signaling machineries in HFpEF remain at large in humans. The ratio of NAD^+^ to NADH decreased in the hearts of patients with T2D, while NAD^+^ is required for metabolic oxidation (Atila Uslu and Uslu, 2022). These findings suggest the possible involvement of altered energy metabolism in pathological hypertrophy.

Interstitial fibrosis is commonly noted in DCM accompanied by changes in a wide variety of cell signaling cascades and extracellular matrix proteins (e.g. formation of insoluble AGEs) (Ren and Ceylan-Isik, 2004; Tayanloo-Beik et al., 2021). In addition, aberrant activation of protein kinases such as calcium/calmodulin-dependent protein kinase type II subunit beta (CAMKII) has been shown to contribute to excitation–contraction coupling defects in T2D. CAMKII may be turned on by the Ca^2+^–calmodulin complex, cAMP, and ROS. Overactivation of CAMKII phosphorylates substrate proteins governing Ca^2+^ handling and fosters LVH, mitochondrial injury, inflammation, and arrythmia (Pyun et al., 2018; Hegyi et al., 2019). It is believed that transient receptor potential channel 3 (TRPC3) and TRPC6 may serve as triggers for the hyperactivation of CAMKII (Klaiber et al., 2010). This is supported by the observation that ablation of TRPC3 or TRPC6 mitigates pressure overload-evoked maladaptive hypertrophy (Seo et al., 2014). Among various potential regulators for TRP channels, stimulation of calcineurin–NFAT is suggested to offer a direct link between gene expression and intracellular Ca^2+^ signaling (Hogan et al., 2003; Molkentin, 2004).

Diastolic function denotes myocardial relaxation capacity following a normal heartbeat (Gaasch and Zile, 2004). The relaxation period includes active and passive phases at both cellular and tissue levels (Borlaug and Kass, 2006). Upon excitation, intracellular Ca^2+^ is dumped from the intracellular sarcoplasmic reticulum (SR) store with the binding of ryanodine receptor (RyR) to troponin C. Relaxation initiates with the dissociation of Ca^2+^ ions from the troponin complex—prior to SR reuptake and efflux of Ca^2+^ through Na^+^–Ca^2+^ exchange protein (NCX). To lower cytosolic Ca^2+^ in diastole, phospholamban is phosphorylated to disinhibit sarcoplasmic/endoplasmic reticulum calcium ATPase 2 (SERCA2), enabling SR Ca^2+^ reuptake (Xu et al., 2020). This is supported by findings that cardiac overexpression of phospholamban inhibits SERCA2A and SR Ca^2+^ uptake to compromise cardiac function, while phospholamban knockout mice display better Ca^2+^ cycling and myocardial contractility. With the uptake of Ca^2+^ into SR during the diastolic period, reverse-mode NCX is increased at the sarcolemma. Decreased myocardial compliance (muscle stiffening) was due to suppressed cGMP-dependent PKG and hypo-phosphorylation of titin (Obokata et al., 2020). cGMP and its cognate kinase, PKG, are known for the maintenance of vascular and endothelial function. As such, the LV chamber becomes less compliant and fails to dilate properly courtesy of hypo-phosphorylation of titin in diastole. Activation of PKG or protein kinase A (PKA, cAMP-driven and possibly PKG-independent) may thus correct these irregularities and reinstate LV chamber compliance (Sztechman et al., 2018).

Hyperglycemia, a hallmark of T2D, is one of the key causes of endothelial dysfunction (Ren and Ceylan-Isik, 2004; Tayanloo-Beik et al., 2021). Exposure of endothelial cells to high glucose leads to ROS production, while glycosylation inhibits eNOS activation, angiogenesis, and mitochondrial dysfunction. Quenching of NO by AGEs plays a vital role in vasodilatory impairment in T2D. Depletion of NADPH and excessive AGE production may result in elevated permeability of endothelial cells, inhibition of eNOS activity, influence of the coagulation system, and activation of NADPH oxidase and nuclear factor-κB (NF-κB) (Soro-Paavonen et al., 2010). Downregulated insulin signaling is a hallmark of T2D, and other signaling cascade changes occur, including downregulated adenosine monophosphate activated protein kinase (AMPK) signaling and increased PKC and mitogen-activated protein kinase (MAPK) signaling with undesirable effects (Ren and Anversa, 2015; Dillmann, 2019). Downregulation of peroxisome-proliferator-activated receptor-γ coactivator 1α (PGC1α) evokes altered oxidative mitochondrial function in T2D. In plasma from HFpEF patients, metabolomic profiling exhibited altered levels of β-oxidation (Phan et al., 2009). Reduced mitochondrial oxidative metabolism is accompanied by increased glycolysis, although such change may be translated to higher glucose uptake through glycolysis from pyruvate oxidation (Berthiaume et al., 2019). Impaired insulin signaling inhibits glucose oxidation by negative feedback regulation via the Randle cycle.

Microvascular disease is also a common feature of T2D (Zhou et al., 2018). Mechanisms include depressed NO signaling, increased oxidative stress and inflammation, impaired angiogenesis, and other abnormalities. For generality, the main factor is NAD-dependent protein deacetylase sirtuin-3 (SIRT3). SIRT3 knockdown of endothelial cells in mice impairs glycolysis and angiogenesis and is associated with diastolic dysfunction. However, a role for SIRT3 in HFpEF patients is lacking. Compared with healthy controls, patients with HFpEF display a greater product of end-systolic pressure and stroke volume, myocardial blood flow, and myocardial oxygen consumption (Pillai et al., 2010). These changes denote an imbalance between demand and supply with elevated cardiac work in HFpEF with β-adrenergic stimulation.

Nuclear factor erythroid 2-like factor 2 (Nrf2) is a master regulator of oxidative stress with a vital role in DKD (Sakashita et al., 2021). Under unstressed conditions, Nrf2 is ubiquitinated and degraded by proteasomes through Kelch-like ECH-associated protein 1 (Keap1), while Keap1 undergoes various chemical modifications to reduce its affinity for Nrf2 and inhibit its degradation under oxidative stress (Ruiz et al., 2013; Kobayashi et al., 2016). Bardoxolone methyl is a synthetic triterpenoid, which not only upregulates Nrf2 but also retards inflammation, perhaps through additional actions on NF-κB. Currently, bardoxolone methyl is going through clinical trials for type 1 diabetic nephropathy and other renal diseases.

## Treatment options for HFpEF in T2D

It is important to identify and treat potential risk factors and other comorbidities such as hypertension, CAD, atrial fibrillation, and valvular heart disease for HFpEF in T2D (Table 1). Therapeutically, mineralocorticoid receptor antagonists (MRA) were reported to reduce HFH in HFpEF, although the effects on mortality-related outcomes and quality of life remain unclear for MRA (Martin et al., 2018). On the other hand, classical β-blockers display little effectiveness in treating obesity/T2D-associated HFpEF, although larger cohorts are needed. Likewise, angiotensin converting enzyme inhibitors exhibit little or no effect on cardiovascular mortality, all-cause mortality, and HFH (Tomasoni et al., 2019). In the PARAGON-HF Trial using angiotensin receptor/neprilysin inhibitor (ARNI, sacubitril/valsartan), there was little difference in primary endpoints of HFpEF. Nonetheless, significant benefits were noted in two subgroups (women and LVEF ≤ 57%). It is suggested that ARNI may be an effective option for certain HFpEF patients (Jering et al., 2021). Comorbidities are common in HFpEF patients, including hypertension, thus necessitating intensive management for hypertension. The ACC/AHA guidelines recommend a target systolic blood pressure <130 mmHg in HFpEF patients. In addition, observational studies suggest that statins may also offer benefits in HFpEF (Yancy et al., 2017).

There are several mechanisms in T2D with HFpEF, including sodium retention and subsequent volume overload and elevated filling pressure, increased proinflammatory cytokines, impaired skeletal muscle function, and cardio–respiratory uncoupling (McHugh et al., 2019; Kessler et al., 2021; Tadic et al., 2021). Historically, treatment based on the cardiovascular system, with neurohormonal blockade, has been claimed unsuccessful. Specific subgroups of patients with HFpEF and those with combinations of clinical comorbidities, including T2D and metabolic dysfunction, might be more new remedies for T2D with HFpEF.

Of note, the use of new medications and combination therapies for glycemic control in T2D is rapidly evolving (Ceylan-Isik et al., 2008; Kashiwagi et al., 2021; Kessler et al., 2021; Oh et al., 2021; Tadic et al., 2021; Kim et al., 2022). As patients with T2D often have other risk factors, including obesity, hypertension, dyslipidemia, and renal disease, current treatment strategies favor combination therapies, such as anti-hyperglycemic and anti-hypertensive therapy, to retard the progression of HFpEF. A multifactorial approach targeting multiple, if not all, risk factors has displayed better promises compared with glucose control alone. The most potent antidiabetic medications to prevent HF development in T2D include SGLT2 inhibitors, which retard the onset and progression of HF regardless of the presence of T2D or not (Anker et al., 2019; Table 2).

**Table 2:** Clinical trials of SGLT2 inhibitors on T2D patients with HFrEF, HFmrEF, or HFpEF.

SGLT2 inhibitors (year of reporting)	Trial design	Participants	Primary outcomes/ endpoints	Follow-up (average, months)	Population	Reference
**T2D with/without established CVD**						
Empagliflozin	EMPA-REG OUTCOME (NCT 01131676)	T2D with established CVD	CVD death/nonfatal MI/nonfatal stroke	37	*n* = 7020; mean age: 63.1	Zinman et al. (2015)
Canagliflozin	CANVAS (NCT 01032629)	T2D with established CVD or multiple risk factors for CVD	CVD death/nonfatal MI/nonfatal stroke	47.2	*n* = 10142; mean age: 63.3	Neal et al. (2017)
Dapagliflozin	DECLARE–TIMI 58 (NCT 01730534)	T2D with established CVD or multiple risk factors for CVD	MACE/CVD/HFH	50	*n* = 17160; mean age: 63.9	Wiviott et al. (2019)
**LVEF ≤ 40% (HFrEF) with or without T2D**						
Dapagliflozin	DAPA-HF (NCT 03036124)	LVEF ≤ 40% with or without T2D	HFH/CVD	18.2	*n* = 4744; mean age: 66.3	Neal et al. (2017)
Empagliflozin	EMPEROR-reduced (NCT 03057977)	LVEF ≤ 40% with or without T2D	HFH/CVD	16	*n* = 3730; mean age: 66.8	Packer et al. (2020)
Empagliflozin	EMPERIAL-reduced (NCT 03448419)	LVEF ≤ 40% with or without T2D	6 MWTD changed	3	*n* = 312; mean age: 69.0	Abraham et al. (2021)
**LVEF < 50% (40%–49%, HFmrEF) with or without T2D**						
Empagliflozin	EMPERIAL- preserved (NCT 03448406)	LVEF < 40% with or without T2D	6 MWTD changed	3	*n* = 315; mean age: 73.5	Abraham et al. (2021)
Empagliflozin	EMPATROPISM (NCT 03485222)	LVEF < 50% without T2D	Evaluated by cardiac magnetic resonance imaging of changes in LVEDV and LVESV	6	*n* = 84; mean age: 62.0	Santos-Gallego et al. (2021)
Sotagliflozin	SOLOIST-WHF (NCT 03521934)	LVEF < 50% with or without T2D	CVD death/HFH	9	*n* = 966; mean age: 69.0	Bhatt et al. (2021)
**LVEF ≥ 50% (HFpEF) with or without T2D**						
Sotagliflozin	SOLOIST-WHF (NCT 03521934)	LVEF ≥ 50% with or without T2D	CVD death/HFH	9	*n* = 256; mean age: 69.0	Bhatt et al. (2021)
Dapagliflozin	PRESERVED-HF (NCT 03485222)	LVEF ≥ 60% with or without T2D	KCCQ-CS	3	*n* = 324; mean age: 70.0	Nassif et al. (2021)

CANVAS, Canagliflozin Cardiovascular Assessment Study; DAPA-HF, Dapagliflozin And Prevention of Adverse-outcomes in Heart Failure; ECLARE–TIMI 58, Effect on Cardiovascular Events–Thrombolysis in Myocardial Infarction 58; EMPA-REG OUTCOME, Empagliflozin Cardiovascular Outcome Event Trial; EMPERIAL-Preserved, Empagliflozin Outcome Trial in Patients with Chronic Heart Failure with Preserved Ejection Fraction; EMPERIAL-Reduced, Empagliflozin Outcome Trial in Patients with Chronic Heart Failure with Reduced Ejection Fraction; KCCQ-CS, Kansas City Cardiomyopathy Questionnaire Clinical Summary Score; LVEDV, LV end-diastolic volume; MACE, major adverse cardiovascular event; 6 MWTD, 6-minute walk test distance.

### Lifestyle modification

Favorable clinical outcome of exercise training in T2D with HFpEF is reminiscent of that seen in HFpEF individuals (Tucker et al., 2016; Bowen et al., 2018). Several clinical trials have depicted that exercise training improves exercise capacity and quality of life in HFpEF patients (Kitzman et al., 2010; Edelmann et al., 2011; Kitzman et al., 2013). On the other hand, calorie restriction and weight loss may be indicated for obese T2D patients with HFpEF. In general, the benefits of exercise training and lifestyle modification seem to reside outside the heart. In T2D and obesity, myocardial glucose is replaced by uptake of non-esterified FFA as the main energy source. Such substrate switch is closely associated with the onset of LVDD, the effect of which can be alleviated by exercise and lifestyle modification (Ren et al., 2021).

### Diuretics

Patients with HFpEF generally develop fluid retention and volume overload, dyspnea, and severe exertional incapacity, with a more pronounced symptomatic manifestation with T2D. Peripheral impairments, such as skeletal muscle dysfunction, impaired peripheral oxygen delivery, and time-varying dysfunction, may result in reduced exercise capacity in T2D patients with HFpEF. Meanwhile, diuretics are recommended for T2D patients with HFpEF and are usually required to treat symptoms and signs of fluid overload. Diuretics are commonly used in pulmonary arterial hypertension (PAH), supporting the utility of diuretics in reducing risks of hospitalization for HF (Zhao et al., 2019). Most clinical trials on the efficacy of treatments for HFpEF have produced neutral results, although strong evidence supports the benefits of diuretics as effective therapies.

### Phosphodiesterase inhibitors

There is evidence for interference of beta-adrenergic receptor (beta-AR) microdomains in HFpEF, with T2D or obese heart exhibiting altered expression levels of beta-AR, coupled with elevated activity of phosphodiesterases (PDEs) as AMP-hydrolysing enzyme. PDE-5 inhibitor sildenafil is an established therapy for PAH. Compared with placebo, treatment with sildenafil did not lead to an improvement in exercise capacity or clinical status in HFpEF, with depressed myocardial oxygen supply particularly during exercise. The evidence of prominent endothelial dysfunction or deficient cGMP signaling could suggest that interventions targeting the NO–cGMP–PDE pathway may be promising for future treatment.

### SGLT2 inhibitors

SGLT2 inhibitors impose benefits on glucose control, as well as renal and sodium–water-related metabolism and hemodynamics (Mudaliar et al., 2016; Ren and Zhang, 2018), to alleviate clinical symptoms associated with HFpEF (Nakagawa and Kuwahara, 2020; Oh et al., 2021). The main benefits of SGLT2 inhibitors are suggested to be improved arterial stiffness and coronary blood flow by way of natriuresis and glycosuria. Meanwhile, SGLT2 inhibitors may impact myocardium directly through alleviating cardiac afterload, interstitial fibrosis, and energy matrix displacement (Zinman et al., 2015; Mahaffey et al., 2018; Radholm et al., 2018). Beyond the glycemic control, SGLT2 inhibitors are deemed effective in the management of medium- to long-term T2D-associated complications (Triposkiadis et al., 2021). Hyperglycemia impairs cardiac function, leading to compromised glucose uptake by T2D (Ren and Ceylan-Isik, 2004; Ren et al., 2010). The increased level of β-hydroxybutyric acid in response to SGLT2 inhibitors leads to a shift of fuel supply from FFA and glucose to the more energy-efficient ketones. Thus, improvement of metabolic efficiency of the heart and kidney while reducing oxygen consumption may be a potential new direction in the future (Lopaschuk and Verma, 2016). These SGLT2 inhibitor-induced biological effects may support the beneficial hemodynamic effects of SGLT2 inhibitors through increasing cardiac mitochondrial energy output. Empagliflozin, canagliflozin, and dapagliflozin have been shown to inhibit cardiac Na^+^/K^+^ exchanger (NHE), resulting in reduced cytoplasmic Na^+^ and Ca^2+^ and increased mitochondrial Ca^2+^ (Uthman et al., 2018). These effects may have a cardioprotective effect, as increased intracellular Na^+^ and NHE activities are associated with myocardial hypertrophy, exacerbation of HF, and arrhythmias (Tomasoni et al., 2022).

### Metformin

In T2D patients with stable CAD, metformin improved LV diastolic function, although the mechanism of action remains undefined. Metformin was associated with lower all-cause mortality in the subgroup of patients with T2D and HFpEF and poor glycemic control (Lin et al., 2020). The possible explanation of its anti-atherosclerotic effect may be its multiple effects on vascular endothelial cells, smooth muscle cells, lipids, and chronic systemic inflammation (Luo et al., 2019), and results show that long-term metformin administration contributes to good lipid metabolism in T2D HFpEF patients.

### Dipeptidylpeptidase-4 inhibitors

Due to the concern of blood glucose control, dipeptidylpeptidase-4 (DPP-4) inhibitors as the drug for the treatment of HFpEF with T2D attract considerable attention. DPP-4 inhibitors can inhibit the decomposition of glucagon-like peptide 1 (GLP-1) and correct glycogenesis of liver in order to improve blood glucose control. The Saxagliptin Assessment of Vascular Outcomes Recorded in Patients with Diabetes Mellitus Study showed that saxagliptin compared with the placebo group was neutral for the major adverse effect of MACE endpoints on cardiovascular death, nonfatal myocardial infarction, or stroke, but had an increased rate of HF rehospitalization (Scirica et al., 2013). At present, only animal studies have shown that DPP-4 inhibitors may promote myocardial fibrosis in elderly diabetic mice (Green et al., 2015; Mulvihill et al., 2016). However, the relationship between the effects of DPP-4 inhibitors and HFpEF is not clear, which needs to be revealed in the future.

### GLP-1 agonists

There was no significant difference in cardiovascular events and hospital stays between GLP-1 agonists and placebo in the ELIXA and ESXCEL Trials (Marso et al., 2016; Ipp et al., 2017). Importantly, the Evaluation of Cardiovascular Outcome Results (LEADER) as well as Semaglutide in Subjects with T2D (SUSTAIN-6) Trials showed superiority compared with placebo by decreasing the risk of major cardiovascular events (Holman et al., 2017; Muskiet et al., 2018). It is noteworthy that the LEADER Trial showed that liralutide failed to improve the hospitalization rate of HF. The effect of this GLP-1 agonist on HF function showed that liralutide appeared to deteriorate HFrEF. However, the effects of GLP-1 agonists on T2D and HFpEF are not clear.

## Conclusion and future perspectives

Clinical trial data show that the incidence rate and long-term mortality rate of T2D patients with HFpEF are higher than those of non-diabetic patients. Treatments for T2D patients with HFpEF are urgently needed. One of the major burning obstacles for clinical therapeutics of HFpEF is the poorly understood pathophysiology behind HFpEF, making drug development a perplexing task. Several potential therapeutic targets have been identified thus far. However, individual targeting of molecular signaling pathways defined in HFpEF might not effectively restore diastolic function due to the apparent drug toxicity and off-target effects. Therefore, future drug development requires a more comprehensive approach, not only for HFpEF comorbidities but also for classification and phenotypic identification in HFpEF.

## Notes

**Author contributions:** M.A. and X.L. drafted the manuscript. X.W., J.R.S., W.W., J.G., J.R., and Y.Z. edited the manuscript. All authors approved the submitted version.
